# Overview of mechanism and consequences of endothelial leakiness caused by metal and polymeric nanoparticles

**DOI:** 10.3762/bjnano.14.28

**Published:** 2023-03-08

**Authors:** Magdalena Lasak, Karol Ciepluch

**Affiliations:** 1 Division of Medical Biology, Jan Kochanowski University in Kielce, Uniwersytecka Street 7, Kielce, Poland

**Keywords:** endothelial leakiness, metal nanoparticles, NanoEL, nanotoxicity, vascular permeability

## Abstract

Nanoparticles (NPs) exhibit unique physicochemical properties that enable them to overcome biological barriers and to be considered one of the best materials with anticancer properties. Most of the administered NPs that end up in the bloodstream interact with the endothelial layer. The interaction of the NPs with the endothelium widens the existing gaps or induces new ones in the monolayer of vascular endothelial cells, thus increasing the access to the target sites in the organism. This type of interaction can lead to NP-modulated endothelial leakiness (NanoEL). The most important factors determining NanoEL are the physicochemical properties of the NPs. NP-modulated endothelial leakiness can lead to the discovery of new therapeutic targets and strategies to improve drug delivery through controlling and regulating NanoEL. Nevertheless, the NanoEL mechanism also carries some limitations that result from an incomplete understanding of NP metabolism and toxicity, and the possibility of their participation in the unintended bidirectional vascular permeability, which may contribute to the formation of cancer metastases. In this review we are focusing on the effect of metal and polymeric NPs on mechanism and degree of induction of NanoEL, as well as on the benefits and risks of using NPs that induce endothelial leakiness.

## Review

### Introduction

**Figure 1:**
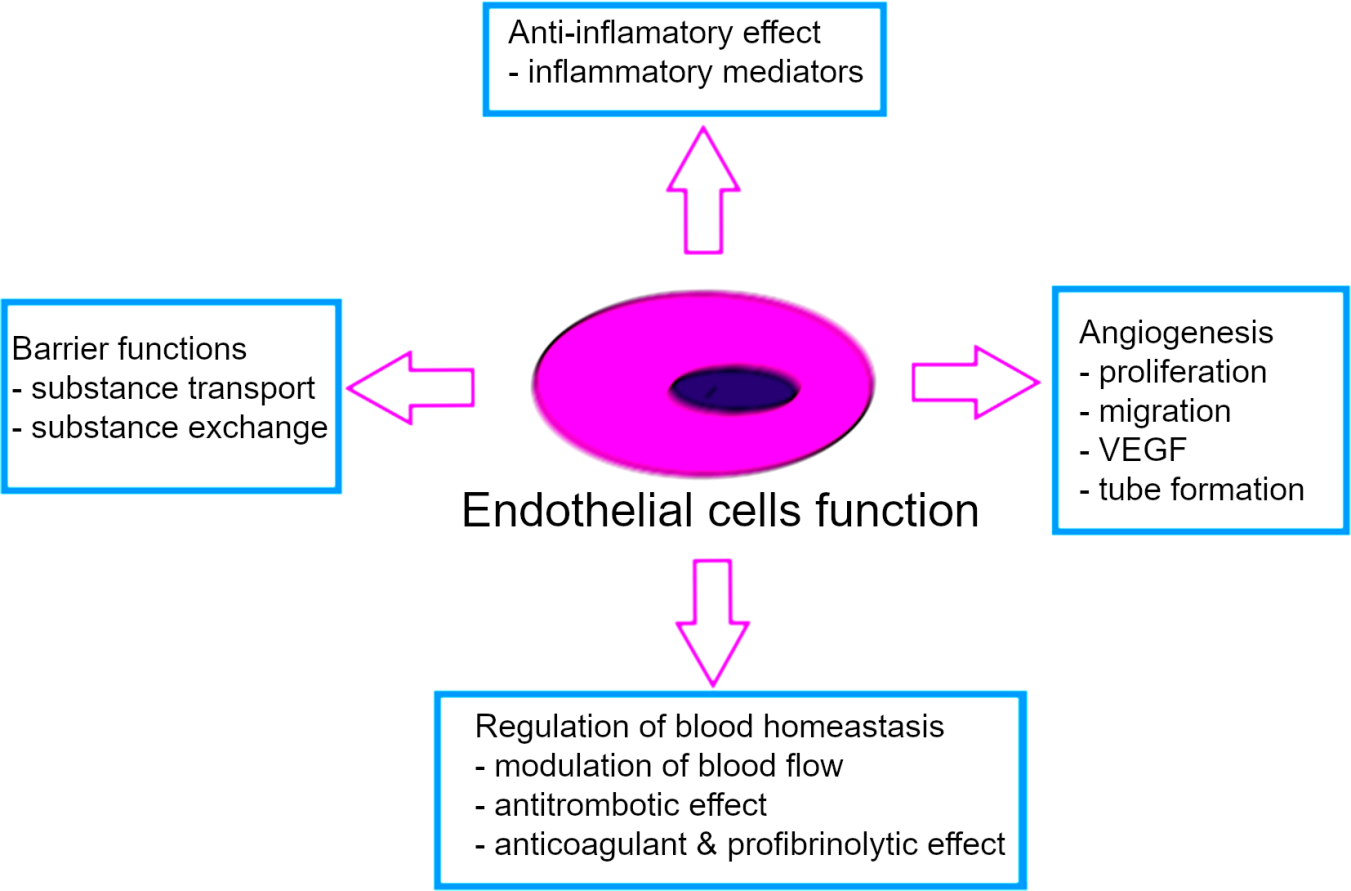
The main functions of endothelial cells.

The vascular barrier is a highly selective boundary between blood and tissues. Its proper functioning is essential to maintaining homeostasis of the whole organism. Formed from mesodermal endothelial cells, the inner lining of blood vessels controls the two-way transport of molecules and ions circulating in the blood and protects the inner tissue environment from harmful and dangerous substances [1–4]. The endothelium secretes vasodilation factors (e.g., nitric oxide and prostacyclin) and vasoconstrictions factors (e.g., thromboxane, prostaglandin, and endothelin-1), regulating vessel tension and, thus, blood flow and pressure [4–8]. The endothelium also produces mediators involved in angiogenic processes (e.g., vascular endothelial growth factor (VEGF) and fibroblast growth factor (FGF)), fibrinolysis (e.g., tissue plasminogen activator (tPA) and plasminogen activator inhibitor (PAI)), and blood clotting processes (e.g., tissue factor pathway inhibitor (TFPI)) [4,6–10]. In addition, endothelial cells are involved in the immune response of the body. Endothelial cells regulate leukocyte diapedesis and produce inflammatory cytokines such as interleukin 6 (IL-6) or tumor necrosis factor-α (TNF-α) (Figure 1). The factors secreted by the endothelium also include angiokines, which affect the parenchymal cells, thus conditioning the functioning of entire organs. Endothelial cells play a key role in the maintenance of the organism’s systemic homeostasis through the many functions they serve. Moreover, these cells are characterized by a unique structural and functional heterogeneity. Morphologically, endothelial cells are mostly flat, spindle-shaped cells in arteries and arterioles, and they are more rounded in capillaries and venules. Their shape is related to the speed of blood flowing through the vessels, where the rapid current stimulates cell elongation [9]. Furthermore, there are primary cilia on the surface of endothelial cells, which act as mechanoreceptors. Cilia pick up extracellular signals and regulate the function of the vascular barrier [3]. The endothelium is also covered by a negatively charged layer of glycocalyx, which has a protective function and is involved in the transport of molecules across the endothelium [11–12].

The degree of endothelial permeability depends on the physiology of the organ. The continuous endothelium is characterized by the lowest permeability. Most organs possess a continuous endothelium, which allows for diffusion of water and small molecules. Transporters, pumps, transcytosis, and ligand–receptor interactions are used to transport larger molecules. Fenestrated capillaries are more permeable than the continuous endothelium and occur in endocrine organs such as the thyroid gland and kidneys. The holes in the cell membrane (fenesters) allow for the selective exchange of larger substances and molecules, for example, hormones, as well as the mass transport of water. Sinusoidal capillaries are characterized by the greatest permeability, and they are found in the liver and spleen. Their increased permeability is caused by the large gaps present between the endothelial cells and in the basement membrane.

The second crucial aspect determining vascular permeability are the intercellular junctions, which condition paracellular transport across the endothelium. There may be tight junctions between endothelial cells, mainly formed by occludin and claudin proteins. This type of connection is most characteristic of the central nervous system (CNS), where endothelial cells form the tightest and the most selective blood–brain barrier (BBB) that provides protection against the penetration of harmful substances and pathogens. Other types of connections include adherens junctions, maintained primarily by transmembrane VE-cadherin, and gap junctions, which are a cluster of transmembrane channels located throughout the vascular system. The intercellular junctions between endothelial cells play an important role in maintaining the monolayer integrity, but they also connect cells in a dynamic and communicative manner [13–14]. Any change in their organization, which results in dysregulated permeability, may lead to pathological conditions such as a heart attack or metabolic or neurodegenerative diseases [1,4,13,15]. Increased permeability of the endothelial layer for particles whose transport is limited under normal conditions is also a phenomenon that is especially characteristic of mature tumors. As the tumor grows, its demand for oxygen and nutrients increases, which causes intensive formation of an abnormal vascular framework (i.e., the angiogenesis process), in which the vessels lose their typical organization and become permeable to molecules of a certain size. This phenomenon is known as the enhanced permeability and retention (EPR) effect [13–14,16].

The tumor vasculature is characterized by a usually incomplete endothelial lining, resulting in relatively large pores (0.1–3 μm in diameter). Such large pores allow for a much higher vascular permeability. Pericyte cells present on neoplastic vessels are loosely associated with endothelial cells and are able to penetrate deep into the tumor, increasing transendothelial permeability [14–16].

In addition to conditions related to the pathophysiology of diseases, other causes of vascular endothelial dysfunction include physiological processes (e.g., aging) or environmental factors such as a high-fat diet. These conditions are accompanied by an increased generation of reactive oxygen species (ROS), which, combined with impaired efficiency of antioxidant systems are one of the most common causes of cell damage including endothelium damage [6,14]. An increased ROS concentration closely correlates with the amount of pro-inflammatory cytokines, whose participation has been confirmed to increase endothelial permeability. Histamine and bradykinin are among the pro-inflammatory cytokines known to increase endothelial permeability. Moreover, modulators that stimulate endothelial permeability include thrombin, angiopoietins (Ang1, Ang2), bacterial endotoxin (LPS), and VEGF (vascular permeability factor). The main mechanism of action of the above modulators is based primarily on the activation of mechanisms controlling the phosphorylation of proteins of intercellular junctions, which in turn opens the cell–cell junctions and creates gaps between the cells [13,17]. The use of appropriate vascular permeability stimulants can therefore be an effective solution in the treatment of diseases whose therapeutic success depends on the effective drug delivery to the target sites through highly selective vascular barriers.

A relatively new method that overcomes the endothelial barrier is the use of nanoparticles (NPs), especially different metal nanoparticles, for example, Au or Ti nanoparticles [18–23]. NPs exhibit unique physicochemical properties that enable them to overcome biological barriers, such as negative surface charge and sizes from 5 to 50 nm. Moreover, most of the administered NPs eventually end up in the bloodstream, which facilitates their interaction with the endothelial layer [18,24–25]. NP-modulated endothelial leakiness (NanoEL) could lead to the discovery of new therapeutic targets and strategies to improve drug delivery through controlling and regulating NanoEL. However, the NanoEL mechanism also carries some limitations that result from an incomplete understanding of NP metabolism, NP toxicity, or their possible participation in unintended bidirectional vascular permeability, which may contribute to the formation of cancer metastases. For this reason, NPs with different physicochemical properties, which can cross the endothelial barrier in a controlled manner with minimal side effects, are being sought [23,26].

This review focuses on the effect of NPs (mostly metal NPs, which are known to lead to endothelial leakiness) on the NanoEL mechanism and the degree of NanoEL induction, as well as on the benefits and risks of using such NPs. Since the rise of nanotechnology and the use of nanoparticles as drugs, researchers have faced new problems, such as nanotoxicity and pathological consequences of their use. Therefore, knowledge about mechanisms and consequences of endothelial leakiness (NanoEL) caused by nanoparticles is crucial to develop effective and safe biocompatible nanoparticles in future.

### NanoEL as a transport route of nanoparticles through the endothelium

#### Main routes to transport NPs across the endothelium

NPs reach the tissues and organs of the body, especially those richly vascularized, through the blood. Thus, the vascular endothelium is the most important permeability barrier for NPs, which is of key importance for their medical applications [5,14]. The ability of NPs to cross the endothelial monolayer depends on both the physicochemical properties of the NPs and the biology and physiology of the vessels themselves [20]. Understanding the mechanism of interactions between endothelium and NPs and the resulting consequences are crucial aspects for the development of effective therapeutic strategies.

**Figure 2:**
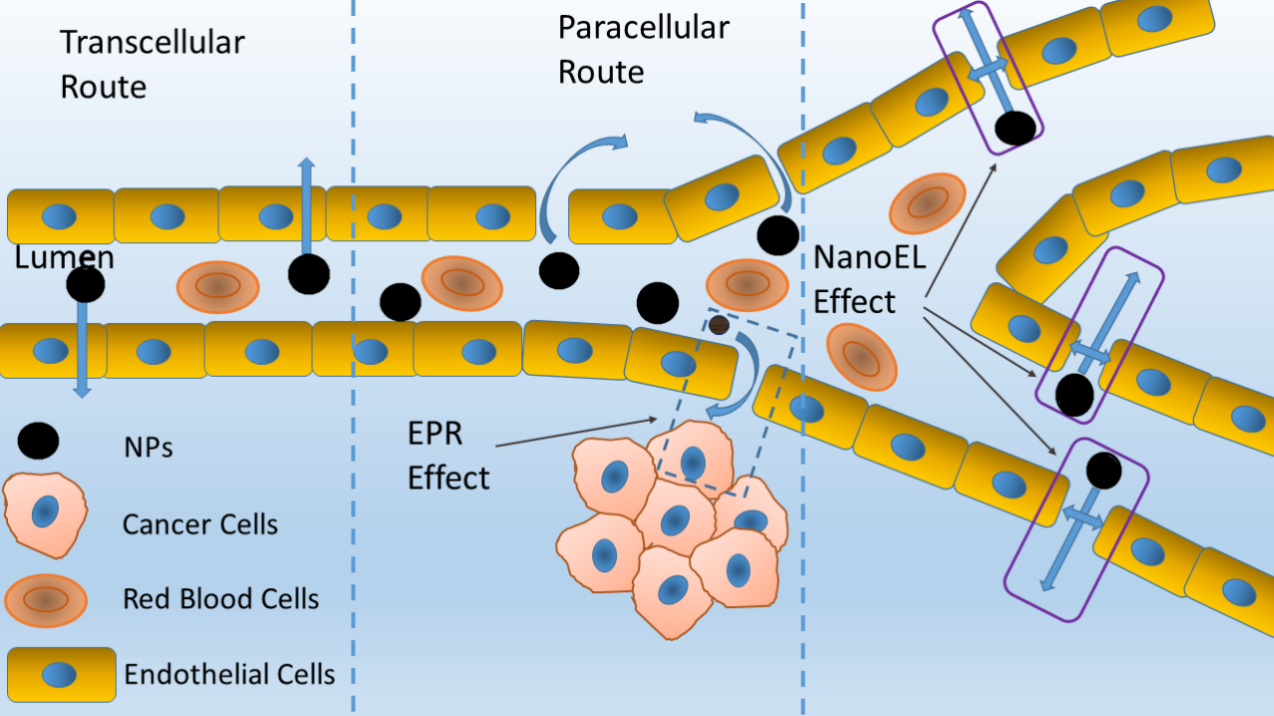
Two main routes to transport NPs across the endothelium, namely the transcellular route and the paracellular route. NanoEL: active transport mechanism of NPs across the endothelium that is unrelated to the transcellular pathway.

There are two main routes to transport NPs across the endothelium, namely the transcellular route and the paracellular route (Figure 2). In the transcellular route, NPs enter endothelial cells through endocytosis and are transported intracellularly [24,27–28]. Recently, the existence of specific endothelial cells involved in the transport of NPs has been reported [29]. Kingston et al. demonstrated the presence of specific vascular endothelial cells in solid tumors that are responsible for the transport of NPs by the transcellular route. They referred to these cells as NP-transporting endothelial cells (N-TECs). Their results suggest that only 21% of tumor vascular endothelial cells, unequally distributed along the blood vessels, participate in transcellular transport. They supported their observations using PEGylated Au NPs of various sizes (15, 50, and 100 nm) in various tumor models. In addition, they characterized the morphology of blood vessels and demonstrated that the vessels with NP-transporting cells were longer and had a greater volume and surface area compared to vessels without N-TECs. Moreover, N-TECs have a different gene expression profile. They are characterized by increased expression of genes involved in transport, such as the PLVAP gene, which mediates microvascular permeability and is involved in the formation of endothelial channels. The discovery of N-TECs creates new therapeutic possibilities, which requires more in-depth knowledge of these cells, their morphology and physiology, and the receptors involved in this transport mechanism [30]. Nevertheless, some limitations resulting from transcellular transport should be considered, because it is an energetically costly, slow, and receptor-dependent mechanism. Moreover, NPs can be retained in the cell and pose cytotoxicity concerns and vulnerability to lysosomal digestion [5,28–29].

The second mechanism, paracellular transport, is based on the penetration of NPs through intercellular gaps. Paracellular transport is an easier route of NP transport than the transcellular route. Nevertheless, the size of the NPs is a key aspect regarding the paracellular pathway [24,27–28]. The width of the gaps between endothelial cells under normal conditions is 2–6 nm. Under pathological conditions, such as cancer, the distance between endothelial cells significantly widens (up to 2000 nm). Most nanomedicine capitalizes on the size of these gaps and relies on appropriately sized NPs to cross the gaps and accumulate at specific sites [31]. In the case of anticancer nanomedicine, an important aspect is the EPR effect, which is a phenomenon characteristic of mature solid tumors. The vascular permeability factors (e.g., VEGF) produced in higher concentrations by cancer cells stimulate the formation of an abnormal vascular structure, which can be used in the passive targeting of nanodrugs. According to this concept, NPs enter the tumor mass through gaps created as a result of increased angiogenesis [26–27]. However, this view turned out to be incorrect, as the effectiveness of the passive delivery of therapeutic agents to the disease sites has been shown to be very low (<1%) [5] and only achievable utilizing NPs less than 100 nm in diameter. In contrast, active targeting strategies involve functionalizing the NP surface with appropriate ligands specific for receptors overexpressed by the cancer cells (e.g., folic acid and transferrin). The combination of the paracellular gap size resulting from the EPR effect and active targeting strategies may increase the efficacy of the therapy. Nevertheless, the accumulation in other, non-targeted organs indicates the existence of a method of NP transport through the endothelium other than EPR [26,28–29,32]. Sindhwani et al. identified an active mechanism based on the transcellular pathway as the dominant mechanism of NP transport through the endothelium. They supported their observations by quantitatively measuring the uptake of 15, 50, and 100 nm PEGylated Au NPs in various mouse tumor models using ICP-MS. Similarly, in a Zombie mouse model, they halted cellular activity using perfusion with a fixative, which allowed researchers to separate the passive and active transport mechanisms [31]. Nevertheless, research currently confirms the existence of a completely different active transport mechanism of NPs across the endothelium that is unrelated to the transcellular pathway.

NanoEL is a mechanism completely independent of receptors and tumor physiology [12,18–19,21,23]. This effect has been observed for various types of NPs including Au [12,21,33–34], Ag [23], Si [20], TiO_2_ [18–19,23], and SiO_2_ [23]. Additionally, NanoEL is responsible for the interaction with adherens junction proteins (e.g., VE-cadherin). The width of the endothelial gaps was ca. 22.5 nm [28]. Researchers observed that the interaction of the endothelium with NPs widens the existing gaps or induces new ones in the monolayer of vascular endothelial cells, thus increasing the access to the organism's target sites [20–21,28]. Researchers reported that NPs can cross the highly selective BBB, which may be important in the case of neurodegenerative diseases. The use of NPs to achieve controlled endothelial leakiness, therefore, creates an opportunity to develop effective therapeutic strategies in various pathological states of the body [35]. However, the leakiness of vessels, up to tens of micrometers wide, also creates an opening for proteins, bacteria, viruses, or the cells themselves. The bidirectional increased permeability of the endothelial layer in the case of tumors may also contribute to faster intravasation and extravasation of cancer cells, which has been confirmed in both in vitro and in vivo studies. Peng et al. demonstrated that the exposure of breast cancer cells to TiO_2_, SiO_2_, and Au NPs significantly accelerates the intravasation and extravasation of tumor cells due to NanoEL and promotes metastasis to new sites [23]. Importantly, NPs can induce endothelial leakiness indirectly, activating the apoptotic pathway or inducing the production of ROS. Moreover, the increased concentration of ROS in the cell contributes to oxidative stress, which in turn also leads to increased vascular permeability [36].

The direct mechanism of induced NanoEL is a relatively easier transport route than the intracellular transport of NPs. However, unlike the EPR effect, NanoEL is a mechanism independent of tumor pathophysiology. Nevertheless, the effectiveness of NanoEL closely depends on the physicochemical properties of the NPs such as size, shape, density, and surface charge [12,19–21].

Moreover, it is worth to mention that the biological effects of NPs to endothelial cells also depend on the microenvironments. The adhesion of proteins and/or nutrient molecules to NPs could change the toxicity of NPs (NP protein corona), and the physiological conditions, such as blood flow and physiological stretch, will also play a role [37–39].

### NanoEL mechanism

**Figure 3:**
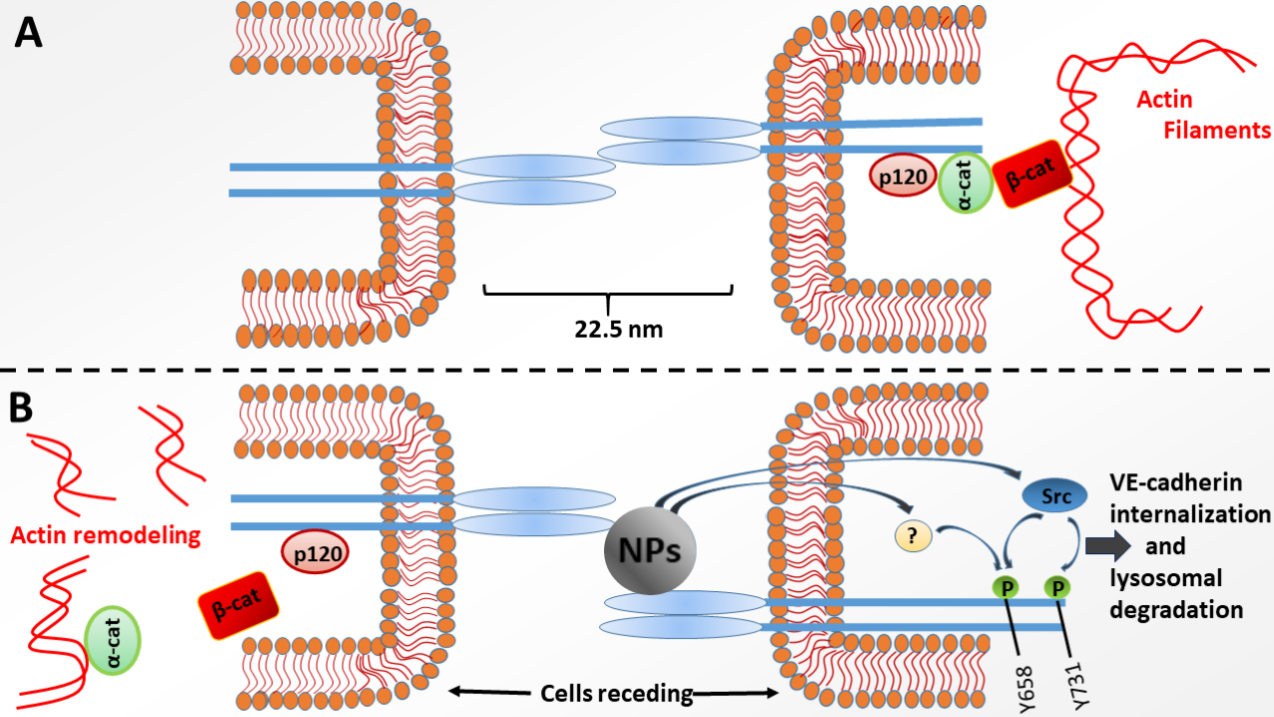
Mechanism of NanoEL. Adherens junctions between endothelial cells are maintained by a complex set of proteins, including the three-component complex of VE-cadherin, p120, and β-catenin. NPs disrupt homophilic VE-cad–VE-cad interactions between endothelial cells, triggering the phosphorylation of intracellular tyrosine residues Y658 (by an unknown kinase pathway) and Y731 (by Src kinase). This causes the loss of stability of the ternary complex of the adherens junctions and the recession of cells.

Adherens junctions between endothelial cells are maintained by a complex set of proteins, wherein the three-component complex of VE-cadherin, p120, and β-catenin, which interacts with the actin cytoskeleton of cells, is of key importance [21,26]. A key role of NanoEL induction is attributed to the extracellular domain of VE-cadherin (VE-cad) located in the intercellular gaps, which are ca. 22.5 nm in size. It is believed that due to the small dimensions of NPs, they can cross these gaps, disrupting the cell–cell junctions and leading to vessel leakiness (Figure 3).

NPs transported paracellularly encounter a size and a charge barrier, which is related to the presence of a negative glycocalyx layer on endothelial cells. Therefore, small (below 100 nm), negatively charged NPs are considered the best NanoEL inducers [5]. In this case, the “bouncing particle” hypothesis proposed by Wang et al. is confirmed, according to which negatively charged NPs target the intercellular gaps through repulsive-sediment interactions with a negatively charged glycocalyx [12]. Using 24 nm Au NPs with different surface charges, Wang et al. supported their hypothesis in in vitro and in silico studies [12]. Also, the concentration of NPs is an important factor regarding controlled leakiness. Au NPs of the smallest size and at the highest concentration significantly promoted cell leakiness and actin reorganization. Increasing the concentration of 18 nm Au NPs from 25 × 10^−6^ M to 100 × 10^−6^ M caused existing intercellular gaps to widen and the formation of new gaps. Tay et al. demonstrated that NanoEL induction depends on the density of NPs, where the effective density of Si NPs ranged from 1.57 to 1.72 g/cm^3^ [20]. The leakage rate increases with increasing nanoparticle density. They also showed that a force of approximately 1.8 nN/μm along the boundaries of VE-cad adherens junctions mediated by cumulative gravity appeared to be the critical threshold force required to disrupt endothelial cells. The leakiness of the endothelium resulting from the action of NPs occurs relatively quickly, usually within an hour after exposing the cells to NPs [20–21,23].

In addition to understanding the key physicochemical properties of NPs, one must also understand the mechanism behind NanoEL. The crucial molecular target of NPs was confirmed to be VE-cad, a protein essential for maintaining the structural and functional integrity of the endothelium [40]. As reported by Setyawati et al., TiO_2_ NPs disrupt homophilic VE-cad–VE-cad interactions between endothelial cells, triggering the phosphorylation of intracellular tyrosine residues Y658 (by an unknown kinase pathway) and Y731 (by Src kinase) [19]. This causes a loss of stability of the ternary complex of the adherens junctions interacting with actin, which are responsible for the shape of the cell. Once the actin remodeling pathway is activated and triggers a change in the shape of the cells, micrometer-sized gaps will form, which are permeable to larger substances and whole cells. The NPs may then be internalized together with VE-cadherin and degraded in lysosomes.

Lee et al. recorded similar observations after examining the leakiness of human skin microvascular endothelial cells (HMVECs) after exposure to citrate-stabilized Au NPs of different sizes and concentrations. Using in vitro, ex vivo, and in silico studies, they confirmed the interaction mechanism of NPs with VE-cad was the phosphorylation of its tyrosine residues stimulated by NPs and actin remodeling, which is crucial for leakiness. It is noteworthy that the computer simulation methods used by Lee et al. showed that Au NPs interact with the extracellular EC1 domain of cadherin [21]. Similarly, the dissociation of the cadherin pair cluster is more effective for localized Au NPs than for NPs evenly distributed throughout the endothelium. Localized Au NPs inhibited the rate of rebinding between adjacent VE-cadherin pairs to a greater extent compared to evenly distributed NPs because of the increasing tensile force, which favored the spread of the crack throughout the VE-cad domain. Lee et al. also suggested that NanoEL is reversible and kinetic, and that a removal of leakiness stimuli triggers the reconstruction of the cadherin bond between adjacent cells. Therefore, NanoEL is a phenomenon unheard of in a tumor-dependent EPR effect. The extracellular NP mechanism of action for NPs including Ag, Au, Si, TiO_2_, and SiO_2_ was also confirmed in other studies, which indicated that it is the most likely method of NanoEL induction.

Endothelial leakiness induced by nanomaterials is a direct mechanism, independent of the receptor. Therefore, to confirm the existence of a new method of controlled blood vessel leakiness (i.e., NanoEL), other indirect effects of NPs must be ruled out. NanoEL was shown to be independent of ROS produced in the cell, oxidative stress, and the apoptotic pathway. The possibility of NP interaction with cell membranes in order to eliminate the participation of transcellular transport was also investigated. In fact, TEM micrographs revealed a small number of 18 nm AuNPs undergoing endocytosis. However, subsequent studies with a cocktail of endocytosis inhibitors confirmed the extracellular mechanism of NanoEL [21].

**Figure 4:**
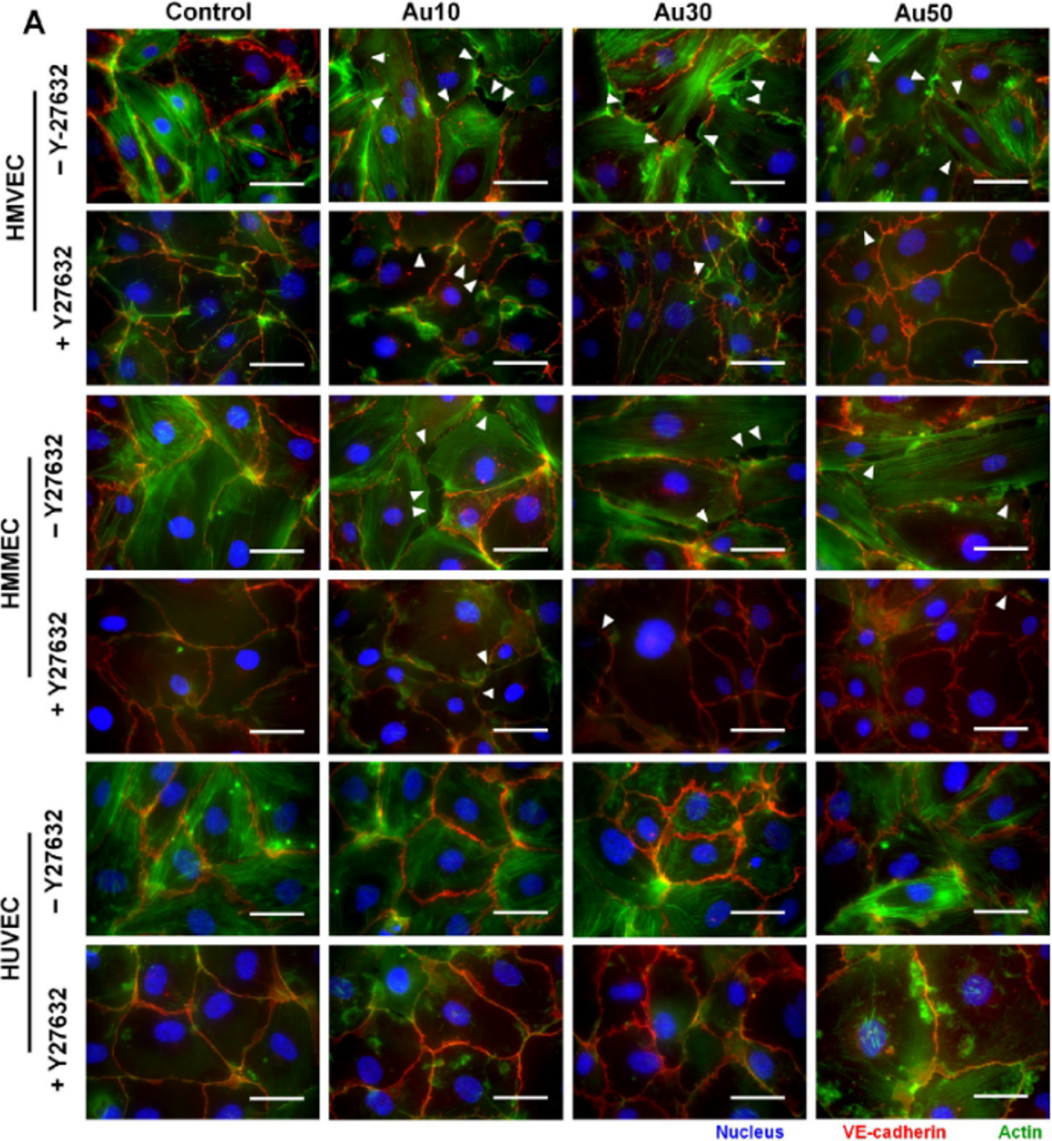
Au NanoEL required actin remodeling. (A) Blocking the RhoA kinase actin remodeling process with Y27632 (10 μM) significantly reduced the gap formation induced by Au NPs (white arrowheads). Green and blue fluorescent signals represent the VE-cadherin and nucleus, respectively. Scale bar: 50 μm. Figure 4 was reprinted with permission from [33], Copyright 2017 American Chemical Society.

There are many aspects to be considered regarding NanoEL, primarily the physicochemical properties of the NPs. Nevertheless, the architecture of the adherens connections and the blood vessels themselves is also a crucial issue. Studying three different types of endothelial cells, Setyawati et al. confirmed a vessel type-dependent NanoEL induction. They observed leakiness induced by Au NPs in HMVECs and human mammary microvascular endothelial cells (HMMECs) and insensitivity to NanoEL induction in the case of human umbilical vein endothelial cells (HUVECs), which form one of the most selective permeability barriers [33] (Figure 4).

Studies on NanoEL are a valuable source of information with respect to the potential future of biomedical applications. Designing NPs with optimal physicochemical properties could enable predicted and controlled leakiness, thus providing a new therapeutic approach that could also be applied in noncancerous applications [41].

### Therapeutic and pathological consequences of NanoEL

The development of nanotechnology creates a number of possibilities in the synthesis of NPs with the desired physicochemical properties, which will determine their various applications. NPs are characterized by a high surface-to-volume ratio, which in turn leads to a large functional surface area. The possibility of combining NPs with various ligands and biologically active molecules, such as nucleic acids, fluorescence dyes, drugs, tumor markers, and proteins, is determined by the stability, biocompatibility, and solubility of such conjugates, which in turn opens up new perspectives in medical applications [16,42]. Nanotheranostic applications use NPs for diagnostic and therapeutic purposes for serious metabolic, cancer, and neurodegenerative diseases. The mechanism of delivery of these compounds to their target sites is primarily based on their transport through the vascular system of the organism. The endothelium is the first and most important barrier to overcome.

As previously discussed, the NanoEL mechanism mainly targets adherens junctions between endothelial cells, which results in a widening of the existing gaps or induces the formation of new ones. The controlled leakiness of the endothelium can therefore increase the access of the transported substances to the target sites and thus increase the effectiveness of targeted therapies. This is particularly important in the case of neurodegenerative diseases, where the effectiveness of most therapies is limited by the highly selective BBB. The width of the tightest connections in the BBB is estimated to be ca. 0.8 nm. Sokolova et al. confirmed the ability of ultrasmall 2 nm Au NPs to penetrate the BBB using a six-cell spheroid model. They reported the ease of the NPs to enter the spheroid and suggested the existence of a mechanism allowing NPs to cross the BBB [35].

The NanoEL mechanism may also play a key role in the treatment of cancer. In fact, NPs can pass the endothelium due to the EPR effect, but transport strictly depends on the stage of the tumor. Hence, the use of the EPR effect may be justified only in the case of mature tumors. Moreover, leaky vessels and heterogeneous blood flow limit its use for therapeutic purposes. By contrast, NanoEL is a mechanism completely independent of the tumor stage. Therefore, NanoEL can be used in the early stages of cancer without requiring the EPR effect. Early diagnosis and treatment when the tumor has accumulated only a few mutations is a key determinant in the effectiveness of the therapy. Moreover, earlier diagnosis and treatment significantly increase the chance of a cure. NanoEL can therefore be used as a new cancer-fighting strategy or to support the EPR mechanism [26,28–29,43].

Despite the vast possibilities that nanotherapy opens, the use of NPs can lead to unforeseen and uncontrolled side effects. As a result, there are only few nanotherapeutic agents approved for clinical use. Nanomaterial-induced leakiness of blood vessels within the tumor may facilitate the penetration of living cancer cells into the bloodstream and to other organs, where they form metastases. Metastases arising from the spread of cancer cells are the main cause of relapse and increased cancer-related mortality. Similarly, the use of NPs in a wide range of consumer products, which may accumulate in the body as a result of routine periodic exposures, could accelerate the formation of metastases in cancer patients. Increased vascular permeability may also contribute to the spread of infections through blood-borne infectious agents and organisms, leading to increased inflammation and tissue swelling due to the extravasation of plasma proteins and blood clotting proteins. There are also reports regarding the toxic effects of NPs on cells. Duan et al. observed that direct exposure of HUVECs to SiO_2_ NPs resulted in oxidative damage induced by ROS, generated by the action of NPs. After 24 h of NP exposure, an increase in cell necrosis and apoptosis was observed. Significant DNA damage and cell cycle arrest at the G2/M point also occurred after NP exposure [44]. Furthermore, the metabolism and removal from the body of NPs are not fully understood and could also pose a threat [15,45].

Despite many limitations, the medical applications of NPs create new perspectives in relation to conventionally used methods. Therefore, nanocarriers with the highest therapeutic effectiveness, regulated pharmacokinetics, known biodistribution, and minimal side effects are being sought. The mechanism of NanoEL shows great potential for future biomedical applications, but a more thorough investigation is still required [5].

## Conclusion

Effective transport of NPs to the target sites of the organism requires the knowledge and understanding of all the possible ways NPs cross vascular barriers. NanoEL is a mechanism with great nanotheranostic potential. The main advantages of NanoEL are the extracellular nature associated with the interaction with VE-cad located in the junctions of the endothelium and the fact that this effect does not depend on the physiology and microenvironment of the tumor. Therefore, NanoEL is a new approach that can be used in anticancer therapies, especially in the transport of medicinal substances to immature and hard-to-reach tumors. Nevertheless, issues regarding the unforeseen side effects of NPs are still under investigation. NanoEL can lead to bi-directional vascular permeability facilitating the intravasation and extravasation of cancer cells. Moreover, with NPs already present in various consumer products, there is a danger of an accelerated and amplified metastatic effect in cancer patients and the accumulation of NPs in unintended organs. Therefore, it is crucial to gain a full understanding of NanoEL and to develop effective and safe inducers, which are NPs that will facilitate access to the target sites and, at the same time, reduce the possibility of side effects and pathology of using them.
